# Experiences of nurses in patient adherence to antiretoroviral therapy in Mpumalanga, South Africa

**DOI:** 10.4102/phcfm.v17i1.4841

**Published:** 2025-04-30

**Authors:** Kabelo Moroko, Zelda Janse van Rensburg, Wanda Jacobs

**Affiliations:** 1 Department of Nursing, Faculty of Health Sciences, University of Johannesburg, Johannesburg, South Africa

**Keywords:** primary health care, antiretroviral therapy, nurses, monitoring, adherence

## Abstract

**Background:**

Human immunodeficiency virus (HIV) is a global health pandemic. Mpumalanga is a province with a high burden of HIV or acquired immunodeficiency syndrome (AIDS). Antiretroviral (ARV) therapy should be initiated for all HIV-positive patients. Monitoring of patients’ adherence to ARV therapy is important to ensure continued viral suppression.

**Aim:**

The study aimed to report on the experiences of primary health care (PHC) nurses in monitoring patients’ adherence to ARV therapy in PHC facilities in Mpumalanga, South Africa.

**Setting:**

Six PHC clinics in a district in Mpumalanga were purposively selected.

**Methods:**

Employing a qualitative, exploratory, descriptive research design, 12 PHC nurses were interviewed in 2023. The data were coded, categorised and clustered into themes and categories. Ethical considerations and measures to ensure trustworthiness were adhered to.

**Results:**

The findings revealed four themes: PHC nurses’ experience in monitoring patients’ adherence to ARV therapy in PHC facilities, experience of external challenges influencing patient’s adherence to ARV therapy, experiences in internal challenges that influence patients’ ARV therapy adherence, and the consequences of non-monitoring and poor adherence.

**Conclusion:**

Non-adhering patients were seen to be the greatest challenge. More awareness regarding the central chronic medicine dispensing and distribution (CCMDD) programme and the development of guidelines on the support of PHC nurses and patients are recommended.

**Contribution:**

The findings of the study may guide recommendations to assist PHC nurses, PHC management and policy makers at large to address challenges in the monitoring and adherence of patients on ARV therapy.

## Introduction

In 2020, 38 million people globally were living with human immunodeficiency virus (HIV).^1^ At the end of 2020, 1.7 million new infections were recorded globally.^2^ In 2020, UNAIDS (Joint United Nations Programme on HIV and AIDS) reported that 5500 new infections were recorded weekly among young women between the ages of 15 and 24 years.^2^ It is estimated that in 2020, about 690 000 recorded deaths were attributed to acquired immunodeficiency syndrome (AIDS)-related illnesses.^2^ South Africa is at the epicentre of the HIV global pandemic.^3^ In 2020, 13% of the South African population was HIV positive. The total number of people living with HIV in South Africa increased from an estimated 3.8 million in 2002 to 7.8 million by 2020.^3^ KwaZulu-Natal in South Africa remains the province with the highest prevalence of HIV/AIDS, followed by Mpumalanga.^4^

Antiretroviral (ARV) therapy is a combination of treatments for patients diagnosed with HIV/AIDS. All people newly diagnosed with HIV/AIDS must be initiated on a lifelong ARV therapy regime. Antiretroviral therapy does not cure the disease but suppresses the HIV replication within a person’s body. The therapy also strengthens the immune system to fight against opportunistic infections.^5^ Antiretroviral therapy significantly reduces HIV-related mortality, especially if viral suppression is achieved.^6^ Antiretroviral therapy is recommended for all patients living with HIV regardless of how long they have been infected with HIV and what their viral load is.^5^

Primary health care (PHC) facilities in South Africa provide a range of services, which include services for HIV/AIDS monitoring, care, prescribing and dispensing of ARV therapy by PHC nurses.^7^ Based on World Health Organization (WHO) recommendations, the South African National Department of Health (SANDoH)^7^ developed guidelines for managing HIV/AIDS in PHC facilities. The guidelines include the steps in monitoring patients on ARV therapy to control the HIV/AIDS epidemic through quality comprehensive treatment and care. The SANDoH set a target of 95% of people knowing their status, 95% of people who tested HIV-positive being on ARV therapy and 95% of people who are on ARV therapy being virally suppressed.^7^ Viral suppression means the virus load is lower than the detectable limit (below 50 copies) or too low to be measured in a standard viral load test.^7^

Monitoring patients on ARV therapy is crucial to promote the sustainability of ARV therapy programmes in South Africa. These programmes include providing quality care at follow-up visits to enhance patients’ adherence to ARV therapy. Patients on ARV therapy should be monitored regularly to determine their clinical response to ARV therapy, their virological and immunological response, and lastly, to manage the side effects of treatment.^7^ Viral load monitoring for HIV-positive patients on ARV therapy must be conducted 6 months from the initiation of ARV therapy, 12 months after initiation, and thereafter, annually.^7^

In the PHC facilities in South Africa, a computer software program called the Three Interlinked Electronic Registers (TIER.Net system) is used to capture data from patients newly diagnosed with HIV. This system is operated by the data team in the PHC facility and is used in capturing newly initiated patient information and continuous linkage to care and monitoring.^8^ The TIER.Net system assists in the electronic monitoring of patients by creating daily, monthly and quarterly reports on HIV-positive patients.^9^ The TIER.Net contains data on clinical events such as ARV therapy initiation, ARV therapy pick-up dates, regimen type and clinic visits, as well as laboratory test results such as CD4 counts and HIV viral loads. With the system, patients are scheduled for appointments and reminded to collect their treatment monthly via telephonic tracing or mobile short messaging service (SMS) a day before the scheduled date.^7^

Patients also need to be monitored monthly after starting ARV therapy.^7^ Viral load testing is recommended as the preferred monitoring approach to diagnose and confirm treatment failure or success.^10^ The optimal goal of ARV therapy is viral suppression.^7^ Adherence to ARV therapy is necessary for patients on ARV therapy to remain in care for optimal clinical outcomes and to improve quality of life.^8^ Patients are therefore monitored periodically in health facilities to ensure adherence to ARV therapy.^7^ However, several factors influence patient monitoring and adherence to ARV therapy.

Although patients are scheduled for appointments for monitoring and collection of ARV therapy, many patients skip their appointments because of unforeseen life circumstances, resulting in poor adherence and an increased risk of ARV therapy resistance.^11^ Missed appointments also result in more Lost-To-Follow-Up (LTFU) patients.^7^ Lost-To-Follow-Up patients are those who have missed visits for 90 days and have not died or been transferred to another clinic. The TIER.Net system generates a missed appointment list, indicating those patients that require tracking and tracing.^12^ Lost-To-Follow-Up patients are firstly tracked and traced through office-based monitoring and thereafter community-based monitoring.

Tracking and tracing by the PHC nurses are done telephonically, but when this fails, patients need to be traced through home visits.^7,13^ Patients are tracked and traced in the community and then encouraged to re-engage in care.^14^ The PHC re-engineering model, established in South Africa in 2010, emphasises community-based care and includes the use of community health workers (CHW) and Ward Based Community Outreach Teams to trace LTFU patients at community level.^15^ Supporting partners, such as non-governmental organisations (NGOs), also assist PHC facilities in tracking and tracing LTFU patients.^14^

There are several internal and external factors that influence monitoring and adherence of patients on ARV therapy. Insufficient PHC staff and inadequate infrastructure at PHC clinics affect the integration of HIV/AIDS services.^16^ Patients are faced with long queues and long waiting periods because of PHC nurses’ increased workload and the lack of infrastructure.^17^ Patients also discontinue treatment and care for a variety of reasons, including mobility (relocation), housing instability, medication side effects and time constraints (being at work and unable to go to the clinic).^12^ All these factors lead to poor-adherence or non-adherence and poor understanding of the importance of ARV therapy and viral suppression.^18^ Poor or non-adherence also relates to myths or beliefs, as some patients discontinue therapy because they believe they have been cured by a higher power.^8^ The non-compliance to ARV therapy promotes the development of drug resistance, which leads to HIV multiplying in the blood system, making the patients susceptible to opportunistic infections, progressing to AIDS and eventually death.^19^

Mpumalanga is a province with a high burden of HIV/AIDS.^20^ Patients receiving ARV therapy place a heavy burden on the South African health system.^7^ Several studies have been conducted on the topic of HIV/AIDS, but limited research is available on PHC nurses’ experiences in PHC facilities on monitoring and adherence of patients on ARV therapy, especially in Mpumalanga, South Africa. This article reports on the lived experiences of PHC nurses in the monitoring and adherence of patients on ARV therapy in Mpumalanga, South Africa. Primary health care nurses monitoring patients on ARV therapy experience several challenges that influence patient monitoring and the adherence of patients to treatment. These challenges are either internally or externally motivated. The aim of this study was to report on the challenges in monitoring patients on ARV therapy experienced by PHC nurses. A view of the experiences of PHC nurses may empower PHC nurses, PHC management and policy makers at large to address these issues and to assist patients to better adhere to ARV therapy by rendering support to patients on treatment. Knowing the experiences of PHC nurses may also lead to the development of guidelines to improve patients’ monitoring and adherence to ARV therapy.

## Research designs and methods

### Study design

A qualitative, explorative and descriptive study was conducted to explore and describe the experiences of PHC nurses in patients’ adherence to ARV therapy in PHC clinics in Mpumalanga, South Africa.

### Study setting

The study was conducted in the Gert Sibande district in Mpumalanga. The Gert Sibande District Municipality is one of the three districts in the Mpumalanga province in South Africa. The district borders on the Kingdom of Eswatini and KwaZulu-Natal, Gauteng and Free State provinces.^21^ The population size of Gert Sibande is 1 135 409 million people with 64.3% of the population made up of people aged 0–34 years.^21^ The mining and manufacturing sectors are strong economic drivers in this district. In 2022, 17.4% of the population in Mpumalanga province was HIV positive. Human immunodeficiency virus prevalence among all ages was highest in Gert Sibande districtt.^21^

The PHC facilities in Mpumalanga render services by trained PHC practitioners, midwife specialists as well as medical practitioners on appointment. The PHC nurses assess, diagnose and prescribe treatment to the patient. The PHC setting offers health care services to the community at district level and is the first level of entry for health care services. The PHC clinics offer services for HIV and AIDS, sexually transmitted infections (STIs), mother, child, women and youth health, expanded immunisation programmes and communicable and non-communicable diseases services. The HIV-positive patients access these PHC facilities to receive their ARV therapy and to be monitored. Antiretroviral therapy coverage in Mpumalanga increased to 81.8% in 2022 from 65.4% in 2017.^22^ An estimated 630 000 people living with HIV in the province were receiving ARV therapy in 2022.^22^ Antiretroviral therapy use among all people living with HIV in the province was 56.4% among adolescents and youth aged 15–24 years, and 83.9% among those aged 25–49 years.^22^ Antiretroviral therapy coverage was 79.8% in urban areas and 79.7% in rural areas.^22^

### Study population, sample size and sampling strategy

Six of 289 PHC facilities from Gert Sibande health district were purposively accessed in this study. All six clinics offered HIV services and monitoring of patients on ARV therapy daily. The proximity of the six clinics allowed the researcher to easily access the clinics. All six clinics presented with high numbers of patients on ARV therapy. At the time of the study, there were a total of 60 PHC nurses working in the six selected PHC facilities in the Gert Sibande region in Mpumalanga where the study was conducted. Two PHC nurses from each of the six PHC clinics were approached and purposively recruited to participate in the study. Twelve PHC nurses from PHC clinics in Mpumalanga shared their experiences in monitoring of HIV positive patients’ adherence to ARV therapy. The PHC nurses were chosen based on their previous experience in monitoring patients on ARV therapy in the PHC setting. The inclusion criteria were PHC nurses working in one of the six selected PHC clinics who were able to understand and speak English well and had at least 1 year of experience in monitoring patients on ARV therapy in a PHC setting. Primary health care nurses that were available from the six clinics, met the inclusion criteria and were willing to be interviewed were included in the study. Because the interviews were listened to and transcribed after each interview, the researcher was able to read and hear the interviews at the same time. After the ninth interview, the researcher realised that the same experiences that were shared by the previous interviewees were heard and read in the last three interviews. After the ninth interview, no new themes emerged. The researcher continued to interview three more participants to ensure data saturation. By the 12th interview, it was concluded that data saturation was reached.

### Data collection

The data were collected at the PHC facility at a pre-arranged date, time and venue that was convenient for the participants. The interviews were conducted between May and June 2022. The data were collected through in-depth, semi-structured, face-to-face interviews. The opening question asked was: ‘How is it for you, as a registered nurse (RN) working in PHC facilities, to monitor patient’s adherence to ARV therapy?’ To elicit a dialogue, the researcher used communication skills and interview techniques, such as probing, active listening, summarising, silencing, clarification and reflection.^23^ Participants were encouraged to talk freely about their experiences in monitoring HIV positive patients’ adherence to ARV therapy after establishing rapport.

A pilot interview was done to determine the central question’s effectiveness and communication skills of the researcher in obtaining the required data and answering the research question. The research question and communication skills of the researcher were deemed effective after the pilot interview, and the data were viewed as valuable and were therefore included in the data set. An electronic audio-recording device was used to record and preserve each participant’s response with permission from the participants. All interviews were conducted in English. Each interview session lasted approximately 45 to 60 min. During the interviews and directly after, field notes were taken by the researcher.

### Data analysis

All the audio-recorded interviews were manually transcribed verbatim. Giorgi’s phenomenological data analysis steps were followed for analysis of the data to code, categorise and cluster the data into themes and categories.^24^ The thematic analysis was done manually according to Giorgi’s steps.^24^ Firstly, the entire transcript was read to gain a sense of the experiences that were shared. Secondly, meaningful units were extracted, by highlighting interesting quotes. Lastly, themes were based on the categories that were derived from the synthesis of the meaningful units of the quotes. The data were manually analysed by the researcher and an independent coder, experienced in qualitative data analysis. The immersion of the researcher in the data was achieved by prolonged engagement in reading the data repeatedly and analysing the verbatim transcripts manually.^24^ The researcher, independent coder and supervisors held a consensus discussion to decide on the themes and categories. Four themes with their categories emerged through an inductive reasoning process.^25^ The themes and categories were supported by the field notes of body language cues, nuances and facial expressions that were taken during and after the interviews.

### Ensuring trustworthiness

Credibility was strengthened by asking one carefully designed central research question. Credibility was ensured through prolonged engagement with the data and peer checking with colleagues, supervisors and independent coder, post interviews and analysis. The recordings were listened to, and the researcher, supervisor and independent coder, all experienced in qualitative research read the transcribed interviews to ensure credibility by checking themes and categories developed from the data. Interview techniques such as probing questions to explore the participant’s lived experience and engaging with the participants during the interview were used.^25^ The measures of transferability were adhered to by providing a dense description of the research setting and supporting the results by direct quotes from the participants. An extensive literature control was done, and the demographic data of the participants were clearly and comprehensively reported on. Cross-checking of the field-notes and the transcriptions as well as consensus discussion with the independent coder were done to ensure credibility. To ensure reflexivity, the researcher kept a personal diary to document his own perceptions and feelings on the topic, and to reflect on the research process during the study. Data were transcribed verbatim directly after each interview and the interviews were analysed independently by the researcher, an independent coder as well as the supervisors. After reflection by the researcher on the process of the research followed, the researcher felt confident that this process adhered to the requirements of trustworthiness and that the analysis was free of any bias.

### Ethical considerations

Ethical clearance to conduct this study was obtained from the University of Johannesburg Research Ethics Committee (No. REC-1203-2021) and the Higher Degrees Committee (HDC-01-87-2021) at a University in Gauteng, South Africa. Permission was also granted from the Mpumalanga Department of Health (MP_202109_002) prior to data collection.

The ethical principles of informed consent, autonomy, beneficence, respect for human dignity and justice were adhered to in this study. The participants received information letters that stated the study aim, objectives and potential risks. The participants were informed that their participation was voluntary, and they could exercise autonomy in their decision to participate or not. The participants had the right to withdraw from the study at any point of time without any negative consequences. The participants gave written consent to participate in the study and for the audio-recordings of the interviews. To date, none of the participants indicated their wish to withdraw from the research. Should any of the participants experienced any psychological discomfort before, during or after the interviews, they were asked to inform the researcher immediately and would have been referred for appropriate psychological counselling. No psychological discomfort was reported before, after or during the interviews. Confidentiality was ensured in the study using a participant code (e.g., P1) to protect the participants’ real identity. Privacy was ensured by conducting the interviews in a private venue, date and time chosen by the participant.

The researcher, supervisors and independent coder were the only ones with access to the audio-recordings and the transcriptions. The audio-recordings and the transcriptions were stored in a password-protected file on a password-protected laptop of the researcher. The password was only known by the researcher. The consent forms were scanned and electronically stored in a separate password-protected file to prevent bridging the devices. As per the University institutional policy, the audio-recordings and the transcriptions will be destroyed after 2 years.

## Results

### Demographic data of the participants

The demographic data of the participants are presented in Table 1. The participants’ demographic information included the participant’s, age, gender and number of years of experience working as a PHC nurse.

**TABLE 1:** Demographic data of the participants.

Participant number	Age (years)	Gender	Years of experience as a PHC nurse
P1	29	Male	9
P2	26	Female	4
P3	30	Female	4
P4	40	Female	12
P5	32	Female	8
P6	32	Female	9
P7	38	Female	6
P8	38	Male	8
P9	38	Female	5
P10	36	Female	6
P11	25	Male	3
P12	33	Male	5

PHC, primary health care.

### Themes and categories

By the 12th concluded interview, data saturation was reached as no new themes and categories emerged. Four themes (Table 2) discussed in this article are: PHC nurse’s experience in monitoring patient’s adherence to ARV therapy in PHC facilities, PHC nurse’s experience of external factors influencing patient’s adherence to ARV therapy, PHC nurse’s experiences in internal factors that influence patient’s ARV therapy adherence and the consequences of non-monitoring and poor adherence by patients on ARV therapy.

**TABLE 2:** Emerging themes and categories.

Themes	Categories
Theme 1: PHC nurse’s experiences in monitoring patient’s adherence to ARV therapy in PHC facilities	Experiences in electronic monitoring Experiences in office-based monitoring Experiences in community-based monitoring
Theme 2: PHC nurse’s experience of external factors influencing patient’s monitoring and adherence to ARV therapy	Health care system and human resource factors influencing ARV therapy monitoring and adherence Health care professional’s influence in ARV therapy monitoring and adherence
Theme 3: PHC nurse’s experiences of internal factors influencing patient’s ARV therapy monitoring and adherence	Social factors influencing ARV therapy monitoring and adherence Personal factors influencing ARV therapy monitoring and adherence
Theme 4: Consequences of non-monitoring and poor or non-adherence by patients on ARV therapy	Resistance to ARV therapy and deterioration of the patient’s health

PHC, primary health care; ARV, Antiretroviral.

### Theme 1: Primary health care nurse’s experience in monitoring patient’s adherence to antiretroviral therapy in primary health care facilities

Participants reported that the monitoring of patients on ARV therapy is a vital part of the prevention of non-adherence. Patients need to be monitored monthly. Participants reported that the monitoring process is three-fold and shared their experiences regarding electronic monitoring, office-based monitoring and community-based monitoring.

#### Category 1: Experiences in electronic monitoring

In the PHC facility, computer software (TIER.Net system) is used to capture data from patients electronically. This system is operated by the data team in the PHC facility and is used in capturing newly initiated patient’s information and continuous linkage to care and monitoring. The participants shared their experience in the electronic monitoring of patients as follows:

‘Okay we monitor them on a monthly basis in the facility, we give them appointment[*s*] monthly to follow up on their treatment and the appointment system works both in paper-based register and also the computerised, whereby the data capture will capture the newly initiated patients on tier.net system.’ (P2, 26 years, female)

‘It is a clever system that we are using, it knows when the patient should be coming back.’ (P4, 40 years, female)

Participants further stated that even though all patients receive an appointment for their follow-up visit, they are also reminded a day before via a cell phone call or SMS of their appointment. The participants explained:

‘Well, the reasons of reminding them is obviously to make sure that they are on treatment, they don’t default.’ (P1, 29 years, male)

‘We have supporting partners who are helping us with that, they will be managing the appointment of the patients in conjunction with tier.net.’ (P3, 30 years, female)

The participants experienced the electronic monitoring system as a good way to monitor patients and to ensure that patients attend scheduled follow-up visits. The participants mentioned:

‘Yes. It is working very well in such a way I know that when to expect this patient, whereby we have our appointment list that we are using.’ (P6, 32 years, female)

‘… [*S*]o basically, we have given them monthly appointment so that they will come in the facility monthly, we have a data capture[*r*] that will use the system of tier.net for appointment which they see by generating [*off the*] list from this system, all patients on ARVs are captured on the system to be able to see what’s needs to be done daily so this also help us with the appointment and how to manage them.’ (P12, 33 years, male)

#### Category 2: Experiences in office-based monitoring

Patients on ARV therapy who do not honour their appointment dates are seen as LTFU patients. Lost-To-Follow-Up patients are individuals on ARV therapy who have either missed an appointment early, after initiation of treatment (7–14 days), late (14–28 days) or thereafter (after 90 days). Patients who missed appointments need to be traced and be encouraged to come for a visit. The participants mentioned:

‘Yes, we have a list and our data capturer help us with that one, we have a list that each and every day we tick that the patient missed their date, and we trace them.’ (P3, 30 years, female)

‘Yes, we have a data capturer who will print out the list of all the clients who have missed appointments and then we take it from there. We need to trace these patients and have them come for an appointment.’ (P7, 38 years, female)

Patients are called and reminded of their missed appointment and a new appointment is scheduled. The participants mentioned that this can be a tedious process:

‘Yes, we call to remind them to come to the clinic if they missed their appointment. This takes us very long to phone them one-by-one.’ (P1, 29 years, male)

‘Then it’s when we phone them to come back to their treatment. We need to do this so that they come back, it is a very tedious job.’ (P5, 32 years, female)

#### Category 3: Experiences in community-based monitoring

Tracking and tracing are the most important aspects of retaining patients to care and preventing LTFU. The National Department of Health (NDoH) initiated services such as mobile clinics and ward-based PHC outreach teams (WBPHCOTs) as part of re-engineering PHC services to ensure that patients on ARV therapy receive integrated services directly at the household and individual level within a catchment area. The participants shared:

‘If the patient is unavailable on the call, we work with CHW (Community Health Worker) who are from the area that the client may be residing, we send them to the patients place to find out if maybe the phone is not working or if is no more or moved and if found they’re referred to the clinic.’ (P1, 29 years, male)

‘… [*W*]hich the supporting partners also at times have RNs which they do home visit to the patients by giving them treatment at home, so that is only possible if they find them at home.’ (P10, 36 years, female)

‘… So, what happens is that the home CHWs they visit them at their home as per address and sometimes the NGOs staff they pack their medications and deliver them at their home if they are successful in tracing.’ (P11, 25 years, male)

### Theme 2: Primary health care nurse’s experience of external factors influencing patient’s monitoring and adherence to antiretroviral therapy

Participants experienced several external challenges related to monitoring patients on ARV therapy. These external factors are related to the health care system, human resources and health care professionals.

#### Category 1: Health care system and human resource factors influencing antiretroviral therapy monitoring and adherence

A high volume of patients that need to be monitored creates a high workload for the PHC nurses. Long waiting times at the PHC facilities and shortage of staff are main contributors for patients not keeping to their appointments. Even when there are enough staff, there are often not enough consultation rooms in the clinics for each PHC nurse to consult with the patient on their own. The participants shared:

‘Overcrowding in the facilities with less staff makes it difficult for the community and patients to honour their appointment.’ (P4, 40 years, female)

‘Waiting period is mostly the contributor as we are serving the community very demanding, the department have challenges, just do the best you can do at the point in time.’ (P7, 38 years, female)

‘Mhmm we are experiencing workload with a lot of patients in the facilities, and in this facility, there is not enough space for each RNs to work, sometimes we work in pairs, which prolong the queue.’ (P4, 40 years, female)

The high volume of patients that need to be seen by the limited number of staff, especially those specifically trained in HIV, cause patients to wait long periods before they can be seen, which influences the service delivery. The participants shared:

‘Shortage of staff is the problem in my facility as we are short staffed which contribute to patients having less interest in honouring their appointment due to long waiting time.’ (P6, 32 years, female)

‘… We are burdened mostly because we usually have overflow of patients with minimal staff which makes it difficult for us to follow up and monitor those patients holistically.’ (P10, 36 years, female)

#### Category 2: Health care professional’s influence in antiretroviral therapy monitoring and adherence

The participants further stated that short courses such as Nurse-Initiated Management of ART (NIMART) are offered for RNs. However, because of the shortage of RNs in PHC facilities, sending RNs for this training increases the burden on the already short-staffed facilities. One participant indicated:

‘In our facility the nurses trained for HIV course (Nurse initiated management of antiretroviral therapy, NIMART) are the one most managing HIV patients and only few of them are trained which makes it difficult for us to see patients when they are off.’ (P2, 26 years, female)

### Theme 3: Primary health care nurse’s experiences in internal factors that influence patient’s antiretroviral therapy monitoring and adherence

Patients often do not adhere to their treatment or do not honour their appointment dates because of social or personal factors. Some patients relocate without notifying the PHC facility. When some patients receive their results that indicate viral suppression, they assume there is no longer a requirement for them to attend the clinic for treatment. Patients often do not understand that viral suppression is attributed to the ARV therapy but rather believe that they do not need to take medication anymore.

#### Category 1: Social factors influencing antiretroviral therapy monitoring and adherence

Social problems include factors such as living far from the PHC facility and not having money to travel to the PHC facility to collect their ARV therapy. Patients also relocate and don’t inform the PHC facility. The participants mentioned:

‘Monitoring of patients is difficult at times with poor adherence as mostly don’t honour their appointment and default on treatment, poor adherence due to social problem.’ (P3, 30 years, female)

‘Sometimes they relocate to other areas without notifying us in the facility which increase in number of lost to follow up, they poorly adhere to treatment and don’t honour their appointment, social problems contribute more.’ (P12, 33 years, male)

#### Category 2: Personal factors influencing antiretroviral therapy monitoring and adherence

Patients often do not adhere to their ARV therapy because of various myths, personal beliefs or not disclosing their HIV status to their partner or family. The participants mentioned:

‘Another challenge is the issue of non-compliance, for example the patients they just decide that they will take treatment the way they want at their own time.’ (P11, 25 years, male)

‘Poor adherence of patients on treatment because of myth or misunderstanding, because most of the patients they are compliant on treatment on start until they are virally suppressed then they start to poorly adhere thinking that they’re now off the hook.’ (P5, 32 years, female)

### Theme 4: Consequences of non-monitoring of poor adherent or non-adherant patients on antiretroviral therapy

Participants shared that the non-monitoring of poor adherent or non-adherant patients on ART could result in patients becoming resistant to their medication. These patients may experience a deterioration in their health and face death.

#### Category 1: Resistance to antiretroviral therapy and deterioration of health

Non-adherence on ARV therapy leads to ARV drug resistance. Resultantly, antiretroviral therapy becomes ineffective, patient’s viral load increases and ultimately their health deteriorates. The participants shared:

‘…Yeah, because the default on treatment they might develop resistant and even their health deteriorates because of the virus.’ (P1, 29 years, male)

‘It becomes a challenge such as defaulting treatment or being resistance as they end up progressing into losing their life which in this era HIV is no longer the killer disease as there is treatment, it is sad.’ (P12, 33 years, male)

## Discussion

The findings of the study revealed that the PHC nurses found the electronic monitoring of patients in ARV therapy as functional. The TIER.Net system was found valuable for recording of ARV therapy initiation, ARV therapy pick-up dates, regimen types and clinic visits, as well as laboratory test results such as CD4 counts and HIV viral loads. The system was also found valuable in reminding patients of their appointments by sending an SMS to them the day prior to their visit. It has been reported that the TIER.Net system is a good system for delivering daily, weekly or monthly reports of the monitoring and adherence of patients on ARV therapy.^9^ Although the TIER.Net system is available and functioning well, the PHC nurses still experienced that patients often miss their appointments and do not adhere to treatment because of missed appointments.

The participants reported that patients on ARV therapy needs special monitoring, on a regular basis. The NDoH^7^ concur with this finding. Although the NDoH has standard operating procedures (SOPs) to outline the process and procedure for delivering effective, sustainable and quality HIV services, it is evident that patients still default on treatment.^7^ Assessing ART adherence to identify the risk of virological failure is a significant challenge.^26^ It is critical to suppress the viral load for patients on ARV therapy to prevent disease progression and transmission of the virus.^10^

The goal of ART is for 95% of HIV patients on ART therapy to be virally suppressed, which means 95% of HIV-infected patients must have a viral load of 50 copies or less.^10^ Viral load testing is recommended as the preferred monitoring approach to diagnose and confirm treatment failure or success. To do viral load testing, patients must visit the clinic on their scheduled visits.^26^

The participants reported that patients on ARV therapy often have personal problems that prevent them from attending their follow-up visits. Personal problems often have an impact on the level of treatment coverage attained, which affects adherence to treatment.^27^ The participants shared that although patients are scheduled for appointments to collect their ARV therapy, many skip their appointments because of unforeseen life circumstances such as not being able to leave work or having to attend to their children.

The participants shared a concern that patients that do not meet their appointments ultimately result in LTFU patients. These patients then need to be tracked and traced to encourage them to re-engage in monitoring and care. Patients on ARV therapy often miss appointments which leads to ARV therapy resistance.^11^ The PHC nurses mentioned that patients that missed appointments then needs to be phoned individually and described this as a very tedious process. When phone calling fails, patients need to be tracked and traced in the community an even more time-consuming task. The PHC nurses mentioned that they often rely on the CHWs, WBPHCOT and NGOs to assist with community tracking and tracing.^11^ The use of supporting partners assisting in tracking and tracing LTFU patients is vital for increasing HIV retention and it is a successful tactic to get the patients back to care.^13^ The challenge with community monitoring is the stigma related to HIV/AIDS and patients often feel embarrassed when they are tracked at their homes. There is often a stigma related to HIV/AIDS and many patients do not disclose their HIV status, not even to their family members.^28^

The participants go on to state that there are several factors that influence monitoring and adherence of patients to ARV therapy. Staff shortage and poor infrastructure in the PHC facilities are some factors that influence monitoring and adherence of ARV therapy. It has been reported that limited PHC staff and inadequate infrastructure affects the proper integration of HIV/AIDS services.^16^ The PHC nurses also stressed that they have an increased workload that cause long waiting times for patients. An increased workload in institutions may have a detrimental effect on service delivery.^16^

A lack of human resources remains a challenge in PHC facilities and has a negative impact on service delivery.^16^ It has been suggested that an amplification of human resources is needed to condense workloads, as human resource constraints influence and affect the monitoring of HIV patients in PHC facilities.^28^

The participants reported several internal factors that influence monitoring and adherence of patients on ARV therapy. Patients often disengage from care for reasons such as relocation, medication side effects and time constraints, such as not being able to leave work to attend the clinic appointment.^29^ The PHC nurses also mentioned that patients have certain beliefs and myths regarding ARV therapy. Many patients believe that when they are virally suppressed, they were cured by a higher power and that it is not the effect of the ARV therapy. It has also been noted that low adherence relates to myths or beliefs, and the authors discovered that some patients discontinue therapy because they believe ‘God’ has cured their HIV.^8^ Non-compliance to ARV therapy then promotes the development of drug resistance, which leads to HIV multiplication in the blood system leading to AIDS and being susceptible to opportunistic infections.^19^

Not all HIV-positive patients on ARV therapy need to receive treatment at the PHC facilities. One recommendation based on the findings of this study is that staff shortages can be addressed by patients collecting their treatment from decentralised pick-up points. The DoH^29^ implemented a central chronic medicine dispensing and distribution (CCMDD) programme. This programme allows clinically stable patients on ARV therapy to receive their treatment at internal or external collection points.^29^ Patients should be made aware of the CCMDD programme by the PHC nurses. To qualify for CCMDD, patients must not be pregnant, have been on ARV therapy for at least 1 year and be virologically suppressed^7^ Patients collect their medication every 2 or 3 months and attend the clinic one to two times a year for prescriptions and annual viral load testing.^29^ While the CCMDD facilitates easy access to treatment collection, social enablers play a significant role in patients’ ARV therapy journey.^30^ These enablers include support and alternative therapies, the lack of which affects adherence to ARV therapy.^30^

Positive social support and family responsibility were discovered to be advantageous to the participants on ARV therapy and should be implemented in the PHC facilities in South Africa. Social support also serves as motivation because ARV therapy patients face stigma and discrimination, which appear to be a barrier to ARV therapy adherence.^30^

### Study limitations

Only PHC nurses from six PHC clinics in Mpumalanga were interviewed in this study. Future, similar research in a larger number of PHC clinical facilities is recommended for the province of Mpumalanga. It might have been likely that crucial information might have been missed because of the sample size. As only 12 PHC nurses from 6 of the 289 clinics were included in the sample, the inclusion of a larger simple size may allow the exploration of additional information on the topic that might have been missed in this study.

### Recommendations

Similar research in various PHC facilities across South Africa is also recommended to expand and include a wider geographical area or provinces in South Africa. The findings from this research could be used to develop a questionnaire and may be used in a survey to quantify the challenges faced by PHC nurses in monitoring patient’s adherence to ARV therapy across South Africa. We recommend that more awareness is created regarding the CCMDD programme to reduce the patient load in PHC facilities. The encouragement of positive social support and family involvement and responsibility is important for patients on ARV therapy. Collaboration with the NDoH as policy makers, and the multidisciplinary health team needs to be in place to offer social support to patients that face stigma and discrimination to ARV therapy adherence. We recommend the development of guidelines to support PHC nurses and patients in monitoring patients on ARV therapy.

We also recommend future studies focussing on personal and social barriers preventing patients from adhering to ARV therapy and determining patient’s need to improve their adherence to ARV therapy.

## Conclusion

The participants found the monitoring of patients on ARV therapy as a vital part of the prevention of non-adherence. Tracking and tracing were viewed as the most important aspects of retaining patients to care and preventing LTFU. Several external challenges related to monitoring patients on ARV therapy were emphasised. These external factors are related to the health care system, human resources and health care professionals. Other factors influencing ART adherence were reported to be social and personal factors. The greatest challenge was viewed as non-adherent patients becoming resistant to ART. The findings of the study suggest ways in which monitoring of patients’ adherence to ARV therapy can be improved. The findings of this study may also guide PHC nurses, PHC management and policy makers at large to address the challenges in the monitoring and adherence of patients on ARV therapy.
